# Data-Driven FTIR Spectroscopy for the Discrimination of Nectars

**DOI:** 10.3390/molecules30204083

**Published:** 2025-10-14

**Authors:** Aleksandra Szaniawska, Justyna Grzeda, Johannes Binder, Andrzej Kudelski, Kamilla Malek, Tomasz P. Wrobel, Andrzej Wysmolek, Katarzyna Roguz

**Affiliations:** 1 Faculty of Chemistry, University of Warsaw, Pasteura 1 Str., 02-093 Warsaw, Poland; jd.grzeda@student.uw.edu.pl (J.G.); akudel@chem.uw.edu.pl (A.K.); 2 Faculty of Physics, University of Warsaw, Pasteura 5, 02-093 Warsaw, Polandandrzej.wysmolek@fuw.edu.pl (A.W.); 3 Faculty of Chemistry, Department of Chemical Physics, Jagiellonian University in Krakow, Gronostajowa 2, 30-387 Krakow, Poland; kamilla.malek@uj.edu.pl; 4 SOLARIS National Synchrotron Radiation Centre, Jagiellonian University, Czerwone Maki 98, 30-392 Krakow, Poland; tomek.wrobel@uj.edu.pl; 5 Faculty of Biology, Botanical Garden, University of Warsaw, Aleje Ujazdowskie 4 Str., 00-478 Warsaw, Poland; k.roguz@uw.edu.pl

**Keywords:** nectar, IR spectroscopy, FTIR, ATR, principal component analysis, chemometrics, *Hedera helix*, *Echium vulgare*

## Abstract

Nectar composition varies across plant species and environments, influencing pollinator interactions and honey quality. Reliable methods for nectar discrimination, however, remain limited. Here, we demonstrate the use of Fourier-transform infrared (FTIR) spectroscopy combined with chemometric analysis to differentiate nectar samples of *Echium vulgare* (*E. vulgare*) and *Hedera helix* (*H. helix*) collected in urban locations. Among eight tested preprocessing strategies, simple approaches such as Savitzky–Golay smoothing or even raw spectra provided the best clustering results. The most discriminative spectral regions were consistently the carbohydrate fingerprint (1200–950 cm^−1^) and the C–H stretching zone (2935–2885 cm^−1^). Mean spectra and PCA confirmed that variability between locations arises mainly from carbohydrate-associated bands, while solvent type, biological matrix, and environmental exposure also affect spectral fingerprints. These results highlight FTIR spectroscopy as a rapid, non-destructive, and robust method for nectar discrimination, with potential applications in food authentication, ecological research, and pollinator–plant studies.

## 1. Introduction

Nectar, a sugar-rich product of flowering plants, serves as a crucial reward for pollinators, playing a pivotal role in plant reproduction and ecosystem dynamics. Its chemical composition is complex and highly variable, reflecting both the physiological state of the plant and the environmental conditions it experiences [1]. Understanding this variability is paramount for ecological studies, agricultural practices, and the quality assessment of derived products such as honey. Nectar typically contains sugars, amino acids, proteins, fatty acids, salts, vitamin C, various secondary metabolites and water [2]. The most common sugars in nectar are sucrose, glucose, and fructose, with average concentration ranging from approximately 10% to 70% *w*/*w* [3]. However, the ratio of these sugars varies among different plant species. Amino acids in nectar are also important for pollinators. They provide essential nutrients that are not present in sugars alone. Some plant species have been found to have a higher concentration of certain amino acids in their nectar, which can make them more attractive to specific pollinators. Secondary metabolites in nectar can include alkaloids, flavonoids, and phenolic compounds. These can have various effects on pollinators, ranging from acting as attractants to serving as deterrents. Nectar volumes can range from less than a microlitre in many bee flowers to several millilitres in flowers pollinated by birds or bats.

A broad spectrum of analytical methods has been employed to study nectar chemistry, including its properties and pollutants. Chromatographic techniques are among the most established. Thin-layer chromatography (TLC) has been applied for analyzing sugar standards and nectar samples, with densitometry enabling quantitative determination of carbohydrates [4]. High-performance liquid chromatography (HPLC) remains a traditional method for quantifying sugars and other analytes, although it requires extensive sample preparation and costly instrumentation [5,6]. Gas chromatography coupled with mass spectrometry (GC-MS) has been widely used for detecting pesticides and plasticizers (e.g., bisphenol A) in nectar [2,7], often following extraction and preconcentration by techniques such as QuEChERS (quick, easy, cheap, effective, rugged, and safe), magnetic solid phase extraction (MSPE) preconcentration, or dispersive liquid–liquid microextraction (DLLME) [8,9]. More advanced approaches such as liquid chromatography–mass spectrometry (LC-MS), tandem LC-MS/MS, and high-resolution Orbitrap LC-MS/MS allow highly sensitive and selective detection of neonicotinoid pesticides, their metabolites, and veterinary drug residues, especially when combined with solid-phase extraction (SPE) or related sample-preparation procedures [10].

Elemental analysis methods also provide important information. Flame atomic absorption and emission spectrometry (FAAS/FAES) have been applied to determine elemental content in honey and nectar [1,11,12]. Inductively coupled plasma mass spectrometry (ICP-MS) and optical emission spectrometry (ICP-OES) enable sensitive quantification of trace and heavy metals, typically following sample digestion (dry ashing, wet ashing with open or closed vessels, often microwave-assisted) or direct dissolution in water [1,2].

Other classical physicochemical methods remain relevant. Refractometry provides estimates of water content and total soluble solids based on refractive index [4]. Titrimetric assays are applied to measure total titratable acidity and ascorbic acid content [13]. The Lane–Eynon method, though nonspecific, quantifies total sugars [6,13]. Spectrophotometric assays detect compounds such as hydroxymethylfurfural (HMF), an indicator of heating or aging, while ash content and electrical conductivity measurements are used to further characterize nectar and honey [4].

Biological and immunological methods also contribute to nectar and honey analysis [6]. Microbiological techniques detect antibiotics such as tetracyclines, while immunoassays and receptor-based tests (e.g., ELISA) allow rapid screening of antibiotic residues in bee products. Imaging and radiological diagnostics have also been applied: diagnostic radioentomology using CT scanning provides non-destructive measurement of density differences within hives, enabling visualization of storage patterns and monitoring of artificial and natural diets.

While these methods yield valuable insights, they are often costly, time-consuming, or require extensive sample preparation or involve sample destruction. Importantly, they are not always suited for rapid discrimination of nectar samples by geographical or botanical origin, which is increasingly relevant for ecological research, authentication of bee products, and protection against adulteration. For this reason, vibrational spectroscopy (FTIR, Raman, NIR), especially when combined with chemometric tools, has gained prominence as a complementary approach, offering a powerful, non-invasive, and rapid means of characterizing the complex chemical fingerprints of biological matrices such as nectar, while enabling authentication of origin, detection of adulteration, and quality control of food products including honeys and fruit-based nectars [13,14,15,16,17,18,19,20,21,22,23,24,25,26]. This method leverages the interaction of IR light with molecular vibrations, providing a rich spectrum of information about various components, including carbohydrates, water, organic acids, and other secondary metabolites [27,28].

Fourier-transform infrared spectroscopy (FTIR) coupled with partial least squares regression (PLSR) has been widely applied to quantify major sugars such as glucose, fructose, and sucrose with high accuracy (R^2^ = 0.97–0.99) and to determine biomarkers such as dihydroxyacetone (DHA), the precursor of antibacterial methylglyoxal in *Leptospermum* nectar [4,5]. FTIR, often in combination with chemometric tools such as PCA, HCA, and PLS-DA, has also been used to classify honeys of different botanical and geographical origins, including Anatolian, Corsican, German, Lebanese and others [18], and to detect adulteration with sugar syrups. More recent approaches have employed advanced multivariate methods such as Common Component Analysis (CCA) to capture subtle regional differences, for example, in Lebanese honeys, while models based on MIR spectra have consistently highlighted the diagnostic importance of the carbohydrate fingerprint region (1150–950 cm^−1^ and 950–750 cm^−1^).

Previous studies have shown that infrared spectral fingerprints capture location- and plant-specific differences, particularly in carbohydrate and C–H stretching regions, enabling effective discrimination of nectar and honey according to origin [4,16,27]. Infrared spectroscopy has therefore become an increasingly important analytical tool in food science, as it is rapid, non-destructive, reagent-free, and can be seamlessly combined with chemometrics. In particular, near-infrared spectroscopy (NIR, 12500–4000 cm^−1^) provides information from overtone and combination bands of C–H, N–H, and O–H vibrations, making it especially effective for on-line and at-line monitoring. Therefore, it supports the development of portable, field-deployable devices. Mid-infrared (MIR), typically acquired with Fourier-transform infrared spectroscopy (FTIR), records fundamental vibrations and thus yields highly specific fingerprints of complex food matrices such as nectar and honey. MIR spectra are dominated by strong O–H stretching of water near 3300 cm^−1^, H–O–H bending at ~1640 cm^−1^, carbohydrate-associated C–H stretching around 2935–2885 cm^−1^, and the fingerprint region from 1500 to 650 cm^−1^, which includes C–O, C–C, and C–H vibrations [4,16]. The 1150–950 cm^−1^ zone is especially diagnostic for carbohydrates, while the anomeric region between 950 and 750 cm^−1^ is highly sensitive for distinguishing floral origins.

Complementary vibrational techniques further extend the analytical potential. Raman spectroscopy, including FT-Raman and surface-enhanced Raman spectroscopy (SERS), provides additional molecular information based on polarizability changes, with minimal interference from water [28]. These methods have been applied for carbohydrate profiling as well as for ultra-sensitive detection of pesticides and antibiotics in honey and nectar [29]. Near-infrared spectroscopy (NIR) has also proven effective for rapid, non-destructive assessment of fruit-based nectars and beverages, enabling robust estimation of °Brix, acidity, and vitamin C content, and supporting classification and adulteration detection through methods such as PLS-DA and SVM [14,30]. Taken together, vibrational spectroscopy and chemometrics provide a versatile toolkit for characterizing nectar and honey, offering accurate quantification of key constituents, authentication of origin, detection of adulteration, and comprehensive chemical fingerprinting that complements traditional chromatographic and elemental techniques.

Overall, these findings demonstrate that vibrational spectroscopy—FTIR, NIR, and Raman—when coupled with chemometrics, is a versatile, non-invasive, and highly informative analytical platform. It can quantify key quality parameters, authenticate origin, detect adulteration, and characterize both nectar and honey across a broad range of floral and geographical sources, complementing but also reducing reliance on slower and more expensive chromatographic or elemental methods.

In our work, we applied FTIR spectroscopy to differentiate *E. vulgare* and *H. helix* nectars collected at different locations within Warsaw, Poland. We performed PCA to probe the landscape of variability coming from different experimental sources. We discuss the influence of the solvent used during the nectar collection on the quality of spectra as well as the differences in the IR spectra of nectar and the flower containing this nectar.

## 2. Results and Discussion

### 2.1. Echium vulgare Nectar

To characterize location-dependent chemical signatures of floral nectars, we analyzed *E. vulgare* samples collected within the Warsaw area. This species was selected as a model because of its wide local distribution and relatively high nectar yield, which facilitated the acquisition of sufficient material for reproducible measurements. Infrared spectra of ten nectar samples representing 7 regions of Warsaw city were measured, preprocessed and evaluated. One of the locations was represented by duplicate samples, one by triplicate samples, while the remaining four locations were represented by a single sample each. The locations are shown in the map in Figure S1 in Supplementary Materials.

Mean ATR-FTIR spectra (±SD) revealed consistent envelopes across all sites with location-specific intensity differences in two windows: C–H stretching (2750–3000 cm^−1^) and the carbohydrate fingerprint (≈1200–950 cm^−1^). Narrow SD bands within sites indicated good intra-location reproducibility, while between-site contrasts were most apparent near ~1100–1000 cm^−1^ and in CH stretches near ~2930–2885 cm^−1^. The most important bands appearing in ATR-FTIR spectra of carbohydrates are listed in Table 1.

In particular, we focused on vibrational modes diagnostic for carbohydrates, which constitute the dominant constituents of nectar and honey. The C–H stretching region (3050–2800 cm^−1^) includes symmetric and asymmetric stretching vibrations of CH, CH_2_, and CH_3_ groups, with the most intense features typically between 2935 and 2885 cm^−1^, directly reflecting the saccharide backbone [31]. The carbohydrate fingerprint region (1200–950 cm^−1^) is dominated by C–O and C–C stretching modes of mono- and disaccharides. Within this range, sucrose exhibits distinctive doublets near ~1150–1050 cm^−1^, while glucose and fructose a single peak at around 1025 and 1053 cm^−1^, respectively (see Figure S2 in Supplementary Materials). The anomeric region (950–750 cm^−1^) is especially sensitive to glycosidic linkages and stereochemistry, with bands near 890 cm^−1^ and 776 cm^−1^ often used to distinguish specific carbohydrate conformations. Additional contributions from the O–H stretching band (~3300 cm^−1^) and the H–O–H bending mode of water (~1640 cm^−1^) are also observed in nectar spectra but can overlap with signals from organic acids and proteins. These diagnostic regions have been widely reported in the FTIR and Raman literature as the most informative for carbohydrate-rich matrices, including honeys and nectars [4,5,13,14].

All preprocessing steps followed a consistent pipeline. First, the spectra were restricted to the spectroscopically relevant regions of 800–1500 cm^−1^ (fingerprint region) and 2750–3000 cm^−1^ (C–H stretching region). An exception was made for Savitzky–Golay smoothing and derivative filters, which, when applied, were computed on the full spectra prior to range selection. After range restriction, baseline correction and normalization procedures (Standard Normal Variate, Multiplicative Scatter Correction, MinMax scaling, or Z-score scaling) were applied as specified for each variant.

Eight preprocessing strategies were tested, including raw spectra (no transformation), Savitzky–Golay smoothing and derivatives, baseline correction, normalization techniques, and their combinations (all variants are listed in Table S2 in Supplementary Materials). Their effectiveness was evaluated using pairwise silhouette scores, which quantify the clustering quality between nectar samples from different collection sites. The silhouette coefficient ranges from −1 to +1: values close to +1 indicate that samples are well assigned to their own group and clearly separated from other groups, values around 0 reflect borderline or overlapping clusters, while negative values suggest potential misclassification.

**Figure 1:**
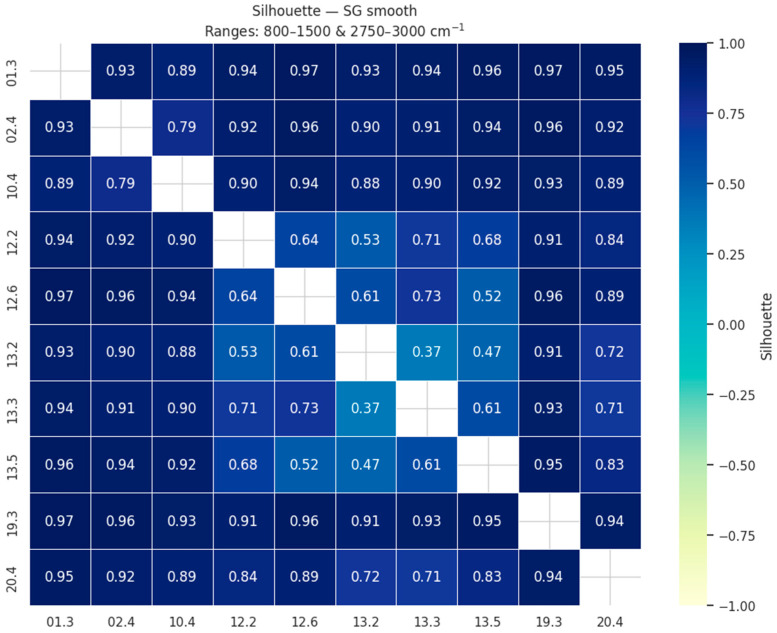
Pairwise silhouette similarity matrix of *E vulgare* nectar samples after Savitzky–Golay smoothing, restricted to the 800–1500 and 2750–3000 cm^−1^ regions. Values close to +1 indicate strong clustering by collection site, values near 0 reflect overlapping clusters, and negative values (not observed here) would suggest potential misclassification. The matrix is symmetric, with both axes corresponding to sample locations (see Figure S1 in Supplementary Materials).

To visualize these results, we constructed heatmaps of pairwise silhouette values (Figure 1). In these matrices, both axes correspond to the sample locations, and each cell reports the average silhouette coefficient for the comparison of the two respective sites. High values (dark blue) indicate that the two locations form compact and well-separated clusters, whereas lighter colors mark weaker separation. The matrix is symmetric, but the full square is shown for clarity. This representation allows one to assess not only the overall clustering quality of each preprocessing strategy but also which specific site pairs are more easily discriminated.

The evaluation showed that the highest clustering quality was obtained for simple approaches, with Savitzky–Golay smoothing (mean silhouette = 0.84) and raw spectra without preprocessing (0.84) ranking at the top, closely followed by Savitzky–Golay smoothing combined with baseline correction and SNV normalization (0.83). Other methods, including baseline correction alone or in combination with MSC, MinMax, or Z-score scaling, yielded slightly lower but still comparable results (≈0.82). More complex preprocessing, particularly the use of Savitzky–Golay derivatives, resulted in markedly lower clustering quality (silhouette < 0.8, and as low as 0.47 for the second derivative). These results indicate that extensive preprocessing is not required for this dataset, and that simple smoothing or even the use of raw restricted spectra is sufficient to achieve clear and robust clustering and separation of samples according to their origin.

**Figure 2:**
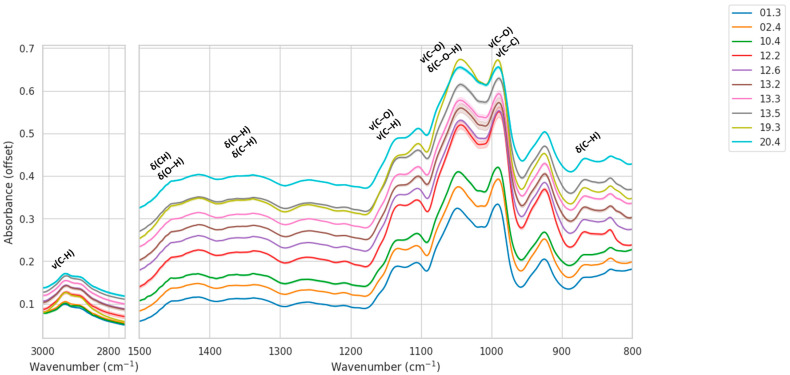
Mean IR spectra (±SD, standard deviation) of *E. vulgare* nectar samples from ten locations after SG smoothing, restricted to 800–1500 and 2750–3000 cm^−1^.

In line with these findings, inspection of the mean IR spectra with standard-deviation envelopes reveals systematic, location-dependent differences across both targeted regions (Figure 2). In the C–H stretching window (2750–3000 cm^−1^), samples from locations 12 and 13 exhibit the highest relative intensities, whereas location 19 shows consistently lower responses, which may be indicative of varying carbohydrate concentration. Within the fingerprint range (600–1500 cm^−1^), the carbohydrate-dominated zone around 1200–950 cm^−1^ displays pronounced variation: locations 12 and 13 present stronger bands near ~1100–1000 cm^−1^, location 10 is intermediate, and locations 01/02 are comparatively weaker. The shaded SD envelopes are narrow, indicating high intra-location reproducibility and supporting the compact within-location grouping quantified in the distance analysis.

Comparable spectral differences have been described in previous FTIR studies of honey and nectar, where variation in carbohydrate vibrations in the 1200–900 cm^−1^ region and C–H stretching bands around 2930–2850 cm^−1^ were identified as key discriminators of botanical and geographical origin. For example, Gok and co-workers showed that differentiation of Anatolian honeys was achieved successfully by ATR-FTIR analysis of the 1800–750 cm^−1^ range [16], while Nickless et al. demonstrated that saccharide composition in *Leptospermum scoparium* nectar could be reliably quantified by ATR-FTIR and related to cultivar-specific chemical signatures [5]. More generally, Wiercigroch et al. emphasized that saccharide marker bands in the 1200–800 cm^−1^ region and CH stretching vibrations between 3050 and 2800 cm^−1^ constitute the principal vibrational fingerprints of carbohydrates in plant materials [31]. Recent systematic reviews confirm that these spectral zones are the most informative for honey authentication and testing for adulteration. Thus, the location-dependent intensity differences observed in our averaged spectra correspond closely with known carbohydrate-dominated features, lending further support to their relevance for nectar discrimination.

**Figure 3:**
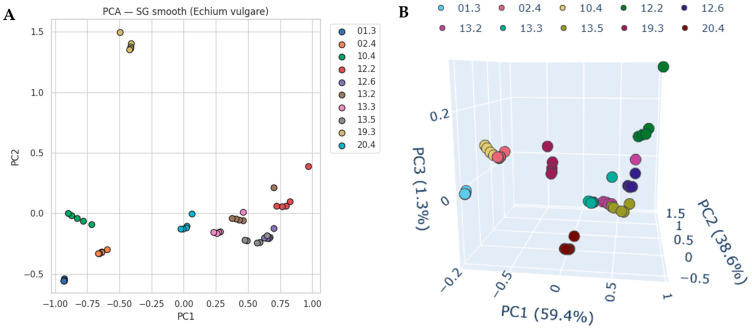
Three-dimensional PCA score plots of averaged IR spectra of *E. vulgare* nectar after SG smooth preprocessing, restricted to the 800–1500 and 2750–3000 cm^−1^ regions. (**A**) PC1 vs. PC2 projection, explaining 59.4% and 38.6% of the total variance, respectively. (**B**) PC1 vs. PC2 vs. PC3 projection, where the third component accounts for an additional 1.3% of the variance.

Principal component analysis (PCA) further highlighted these systematic spectral differences (Figure 3). For the SG-smoothed dataset, the first three components explained together nearly 99.3% of the variance (PC1 = 59.4%, PC2 = 38.6%, PC3 = 1.3%). Samples grouped tightly according to their geographical origin, with most locations clearly separated along PC1 and PC2. Nevertheless, samples from locations 12 and 13 partially overlapped, reflecting their high spectral similarity. The three-dimensional PCA projection (PC1–PC3) provided slightly improved visual discrimination between these two locations, with PC3 capturing subtle differences in the carbohydrate-associated region.

Inspection of PCA loadings plots (Figure S3) revealed that PC1 and PC2 variance is dominated by carbohydrate-associated bands, especially the broad region around 1050–1000 cm^−1^, with notable contributions from bands near 980 and 930 cm^−1^. These features correspond to C–O and C–C stretching in glucose, fructose, and sucrose. PC2 emphasizes similar carbohydrate bands but with slightly shifted contributions. PC3, although capturing only 1.3% of variance, highlights CH-stretching vibrations at 2930–2850 cm^−1^, indicating that even subtle variance in this region helps refine separation of closely related locations.

It should also be noted that not all locations were perfectly separated based on only PCA of total variance. This limitation may be partly methodological: nectar sampling is inherently challenging, as the available volume per flower is small, necessitating manual collection from a variable number of blossoms for each sample. Such variability introduces potential heterogeneity at the collection stage. On the other hand, the data clearly demonstrate that large geographical distances are not required to detect environmental influences on nectar IR spectra. Even within the same city, local conditions—such as differences in shading, soil composition, surrounding vegetation, presence of microorganisms, micro- and macro-organism activity, solar exposure, and local pollution—can shape the spectral fingerprint of nectar. With a greater emphasis on standardized sampling protocols, these subtle yet consistent environmental influences could be captured more systematically, enhancing the discriminatory power of the approach.

### 2.2. Hedera helix Nectar

**Figure 4:**
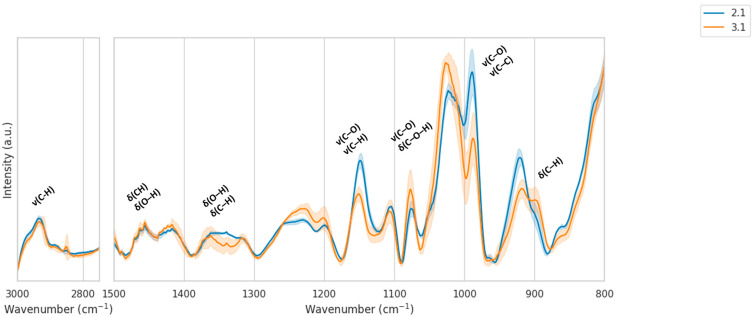
Mean IR spectra (±SD) of *H. helix* nectar samples from two locations after baseline correction and MinMax normalization, restricted to 800–1500 and 2750–3000 cm^−1^.

The next set of nectar samples was collected from *H. helix*. Due to the very low nectar content in the flowers, the diversity of the obtained dataset is considerably reduced compared to *E. vulgare*. As a result, the standard deviation of the IR spectra is higher, which makes spectral differences between the groups less pronounced (Figure 4). In contrast to *E. vulgare*, where the separation of the groups was clearer, in *H. helix* the overlapping of signals can indicate plant’s higher resistance to local environment’s variability. This suggests that *E. vulgare* nectar provides a better-resolved spectral fingerprint than *H. helix*.

All preprocessing steps followed the same pipeline as in the *E. vulgare* dataset. The spectra were restricted to the spectroscopically relevant regions of 800–1500 cm^−1^ (fingerprint region) and 2750–3000 cm^−1^ (C–H stretching region).

Inspection of the mean IR spectra with standard-deviation envelopes (Figure 4) indicates that, in contrast to *E. vulgare*, the spectral variability within *H. helix* samples is higher, reflected in broader shaded regions. This higher variance reduces the clarity of location-dependent differences. Nevertheless, several systematic spectral features are noticeable. In the C–H stretching region (2750–3000 cm^−1^), both locations show overall similar band shapes, though with subtle differences in intensity distributions. In the fingerprint region (800–1500 cm^−1^), the carbohydrate-dominated window (1150–950 cm^−1^) again plays a major role: location 2.1 tends to display slightly stronger absorbance near ~1050–1000 cm^−1^, whereas location 3.1 shows broader, less distinct features, coupled with increased baseline fluctuations. The more pronounced variability in location 3.1 is consistent with its wider SD envelopes, suggesting that nectar composition at this site is more heterogeneous. Part of this variability may also originate from the collection procedure, as rinsing each nectary disc with water or ethanol to obtain sufficient nectar volume could introduce solvent–matrix effects, further contributing to the broader variance observed in *H. helix* spectra (see also Section 2.3).

**Figure 5:**
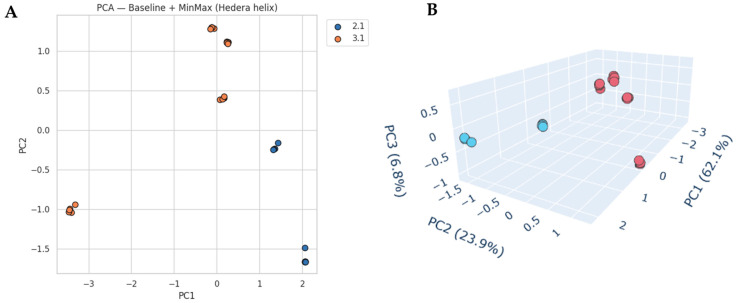
PCA score plots of *H. helix* nectar spectra after baseline correction and MinMax normalization, restricted to the 800–1500 and 2750–3000 cm^−1^ regions. (**A**) 2D plot (PC1 vs. PC2), where PC1 explains 62.1% and PC2 23.9% of the total variance; (**B**) 3D plot (PC1 vs. PC2 vs. PC3), with PC3 accounting for an additional 6.8%.

Principal component analysis (PCA) further highlights these differences (Figure 5). The two-dimensional PCA score plot (PC1 vs. PC2) reveals a clear separation between locations 2.1 and 3.1. PC1 explains 62.1% and PC2 23.9% of the variance, together capturing over 86% of the spectral information. The three-dimensional PCA projection (PC1–PC3) further illustrates this separation, with PC3 (6.8%) adding minor discriminative information. Samples from location 2.1 cluster more tightly, while those from location 3.1 are more dispersed, indicating greater internal variability.

The PCA loadings (Figure S4) confirm that the dominant discriminatory features appear in the carbohydrate-associated fingerprint region, particularly between 1100 and 950 cm^−1^. Additional contributions from the C–H stretching region also plays a role, though to a lesser extent. These findings align with the carbohydrate-dominated nature of nectar IR spectra, as observed also for *E. vulgare*.

Taken together, the results show that while *H. helix* nectar spectra display systematic, location-dependent variation in the fingerprint and C–H stretching regions, the increased variability within samples—particularly at location 3.1—is reflected in the inherent heterogeneity of *H. helix* nectar composition and the practical limitations of collecting sufficient nectar volumes from this species.

The two case studies conducted on *E. vulgare* and *H. helix* nectar samples highlight both commonalities and subtle differences in how FTIR spectroscopy combined with chemometric analysis can discriminate between samples of different geographical origins.

For *E. vulgare*, a larger set of locations was represented, including both single- and duplicate-sample sites. Silhouette analysis demonstrated that straightforward preprocessing or even raw spectra were sufficient to achieve compact clustering within locations and large distances between locations. PCA confirmed this outcome, with most locations clearly separated. Mean spectra revealed systematic differences between locations, especially in carbohydrate-associated bands (~1200–950 cm^−1^) and C–H stretching vibrations (2930–2850 cm^−1^).

For *H. helix*, the dataset was smaller (two locations, each with multiple biological replicates), but the results were consistent with those obtained for *E. vulgare*. PCA revealed that while location 2.1 samples clustered tightly, location 3.1 showed greater internal variability, as evidenced by both the PCA dispersion and the broader standard-deviation envelopes of the mean spectra. The spectral regions most responsible for the discrimination were again the carbohydrate fingerprint region (~1100–1000 cm^−1^) and the C–H stretching region (~2950–2850 cm^−1^), where location 3.1 displayed higher intensity and variability.

Taken together, these analyses provide converging evidence that FTIR, when combined with appropriate preprocessing and multivariate analysis, is highly effective in discriminating nectar samples by different locations, even with small distances, although the number of samples can be crucial to obtain reliable results. Moreover, the PCA loadings suggest that differences between locations are not systematic between species. The results reinforce previous literature findings that carbohydrate- and C–H-associated bands are the primary discriminators of nectar and honey provenance. Importantly, while *E. vulgare* demonstrated robust separation across multiple locations and *H. helix* confirmed the approach in a more constrained dataset, both case studies highlight the reproducibility of the method and its suitability for broader application in nectar and honey authentication.

### 2.3. Significance of the Sample Collection

**Figure 6:**
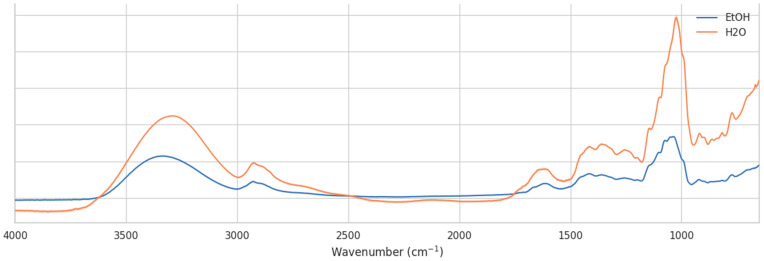
Comparison of FTIR spectra of *H. helix* nectar collected with water and ethanol.

The choice of solvent for nectar sample preparation has a substantial impact on the infrared spectral profile and, consequently, on the interpretation of chemical composition [32]. Figure 6 compares representative spectra of *H. helix* nectar collected in water and ethanol. While the overall spectral envelopes are similar, clear intensity differences and band distortions are observed between the two solvents.

In the carbohydrate-dominated fingerprint region (1200–950 cm^−1^), spectra obtained from water-collected samples display sharper and more intense features, particularly around 1050–1000 cm^−1^, which can be attributed to C–O and C–C stretching vibrations of mono- and disaccharides. In contrast, ethanol-collected samples show lower absolute absorbances and a smoother profile in this range, likely due to partial overlap and hydrogen-bonding interactions with residual water. Similarly, in the C–H stretching region (2950–2850 cm^−1^), water-collected spectra exhibit more pronounced peaks, whereas the ethanol-collected spectra are attenuated.

These observations demonstrate that ethanol as a collection medium may enhance the resolution of saccharide-associated vibrational modes, facilitating more robust chemometric discrimination between samples. However, ethanol also introduces a risk of selective solubility and potential extraction bias, as some polar compounds may be preferentially retained in aqueous solutions. Water collection, on the other hand, minimizes solvent-related chemical interference but may suppress spectral features due to stronger hydrogen-bonding interactions and baseline distortions.

Overall, the comparison highlights the importance of standardized sample collection protocols. Differences between water- and ethanol-collected spectra, suggesting that direct comparison across solvents may lead to artificial clustering or misclassification. For reliable discrimination of nectar samples by location or botanical origin, consistent solvent use is essential, and ethanol may provide an advantage by amplifying the relevant carbohydrate signatures in the mid-infrared region.

### 2.4. E. vulgare flowers

**Figure 7:**
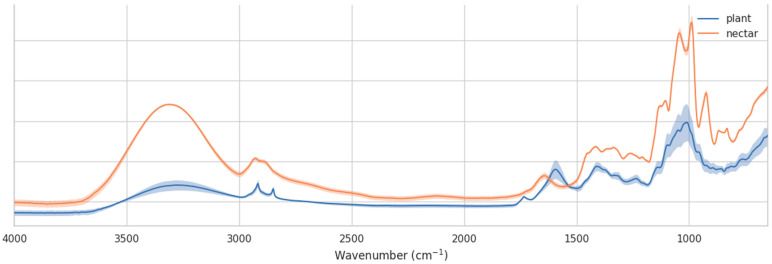
Comparison of FTIR (±SD) spectra of *E. vulgare* nectar and flowers.

The comparative analysis of FTIR spectra from *E. vulgare* nectar (collected in water) and *E. vulgare* floral tissue (Figure 7) highlights the distinct chemical signatures of secreted nectar versus the plant matrix from which it originates. Although both spectra share common vibrational features related to carbohydrate structures, the relative band intensities and overall profiles are markedly different, reflecting their differing biochemical compositions.

In the carbohydrate-dominated fingerprint region (1200–950 cm^−1^), the nectar spectrum displays more intense and well-defined absorptions, consistent with a higher proportion of free sugars (primarily glucose, fructose, and sucrose). The flower tissue spectrum, by contrast, shows broader and less intense peaks in this range, indicative of saccharide moieties bound within complex cell wall polymers such as cellulose and hemicellulose.

The C–H stretching region (2950–2850 cm^−1^) is more pronounced in the nectar spectrum, further supporting the dominance of free carbohydrates and other low-molecular-weight organic compounds (e.g., sugars and organic acids). In the flower spectrum, however, additional bands appear in the 1700–1600 cm^−1^ region, attributable to C=O stretching and aromatic vibrations of phenolic compounds and proteins present in cellular structures. This suggests that flower tissue spectra are more chemically diverse, incorporating contributions from structural biopolymers, lipids, and secondary metabolites.

Overall, these results confirm that nectar spectra are dominated by soluble carbohydrates and present a cleaner fingerprint for chemometric discrimination, even of local geographical or botanical origins. In contrast, flower spectra reflect a more complex biochemical matrix, with significant contributions from structural and defensive metabolites. While flower tissue analysis provides valuable information about plant chemistry, nectar is a more suitable matrix for assessing the chemical cues available to pollinators and for authentication of honey origin.

These comparative analyses across both species further emphasize the importance of matrix and solvent choice in FTIR-based studies of nectar. In *H. helix*, the difference between water- and ethanol-collected samples highlighted how solvent effects can alter spectral intensity and band resolution, potentially influencing chemometric discrimination. In *E. vulgare*, the comparison of nectar with floral tissue underscored that while both share carbohydrate-related vibrational features, nectar provides a much cleaner and more reproducible spectral fingerprint dominated by soluble sugars. Together, these findings demonstrate that reliable authentication and discrimination depend not only on preprocessing and multivariate analysis but also critically on the selection of the biological matrix (nectar vs. plant tissue) and the collection medium (water vs. ethanol).

### 2.5. Artificial Test Samples

To further support the interpretation of chemical differences between natural nectar samples from different localities, artificial model samples were prepared and analyzed by FTIR spectroscopy. These comprised two environments: DIRTY (kept next to the crowded street) and CLEAN (kept in the garden as the reference), with a 40% sucrose solution in water serving as a control.

**Figure 8:**
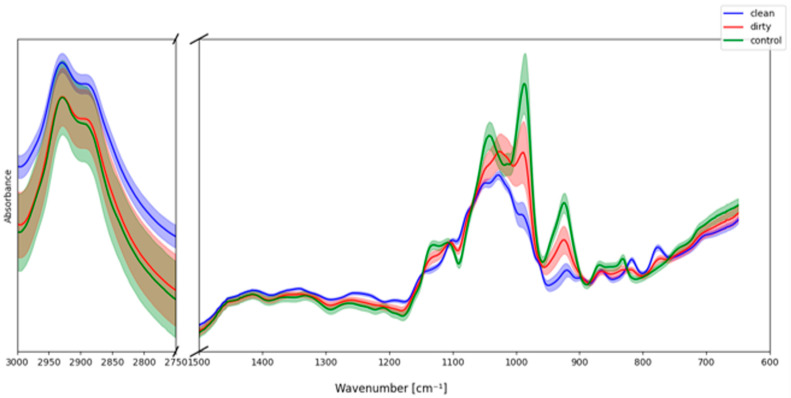
FTIR (±SD) spectra of clean, dirty and control artificial samples. The spectra are limited to the region of 600–1500 and 2750–3000 cm^−1^ for better visibility; the full range spectra are displayed in Figure S5 in Supplementary Materials.

Average IR spectra of the three groups (Figure 8) revealed clear differences, particularly in the fingerprint region (600–1500 cm^−1^). The CLEAN samples displayed sharp and well-defined carbohydrate bands, closely resembling the sucrose control, especially in the 1200–950 cm^−1^ region characteristic of C–O and C–C stretching in sugars. By contrast, the DIRTY samples exhibited broader absorptions and baseline distortions, with reduced band resolution in the range of 1100–1000 cm^−1^, suggesting the presence of additional chemical constituents beyond pure saccharides.

Differences were also evident in the C–H stretching region (2750–3000 cm^−1^). While both CLEAN and control spectra showed typical carbohydrate-associated bands, the DIRTY samples displayed lower intensity and a flatter profile, consistent with environmental modifications or interference. These observations suggest that exposure to different environments can result in detectable chemical signatures in nectar-like matrices, affecting both band intensities and reproducibility.

Overall, the artificial sample analysis demonstrates that environmental conditions can significantly influence the IR spectral fingerprints of nectar analogues. The sucrose control highlights the baseline carbohydrate profile, while deviations in CLEAN and DIRTY samples illustrate how environmental exposure contributes to chemical variability. These findings support the interpretation of natural nectar spectra, where differences between localities may in part reflect environmental inputs beyond the intrinsic plant-derived carbohydrate composition.

These observations from the artificial samples strengthen the interpretation of the natural nectar datasets. The differences between CLEAN and DIRTY samples mirror the location-dependent variability observed in both *E. vulgare* and *H. helix* nectars, where samples collected from distinct environments showed variation in the carbohydrate fingerprint and C–H stretching regions. Thus, the artificial models confirm that part of the spectral heterogeneity in natural nectars likely originates from environmental influences, and they validate the chemometric discrimination of nectar samples by geographical origin.

### 2.6. Significance of Sample Collection

The comparative experiments carried out on nectar samples, plant tissue, and artificial models underline the importance of collection strategy and sample type for FTIR-based analysis. Each choice—solvent, biological matrix, or environmental exposure—can markedly influence spectral fingerprints and, consequently, the interpretation of chemometric results.

For *H. helix* nectar, the comparison of spectra collected in water and ethanol revealed that solvent strongly modulates spectral intensity and resolution. Ethanol-collected spectra displayed sharper and more pronounced carbohydrate-related bands, especially in the fingerprint region (1200–950 cm^−1^) and C–H stretching region (2950–2850 cm^−1^), whereas water-collected spectra showed dampened intensities due to hydrogen-bonding effects and residual solvent background. These findings demonstrate that consistent solvent use is critical, as differences introduced at the collection stage may propagate into chemometric models, potentially leading to artificial clustering.

For *E. vulgare*, the contrast between nectar and floral tissue spectra further emphasized the importance of selecting the appropriate biological matrix. Nectar spectra were dominated by soluble carbohydrates, producing cleaner and more reproducible fingerprints, while flower tissue spectra contained additional features arising from structural biopolymers and phenolic compounds. Although tissue spectra provide insight into plant chemistry, nectar offers the most relevant matrix for studies of pollinator interactions and for authentication of honey origin, as it directly represents the plant-derived sugar pool.

Artificial model samples offered additional evidence of the role of environmental exposure. Spectra from CLEAN and DIRTY samples, compared to a sucrose-water control, displayed noticeable differences despite their common carbohydrate base. The clean samples closely resembled the sucrose control, while the dirty samples exhibited broader bands, baseline shifts, and reduced resolution, consistent with the incorporation of environmental contaminants or additional organic constituents. These results demonstrate that environmental context can introduce variability into nectar spectra, paralleling the location-dependent differences observed in natural nectar datasets of both *H. helix* and *E. vulgare*.

Taken together, these findings highlight that reliable discrimination of nectar samples requires careful attention to sample collection strategy. Solvent, biological matrix, and environmental exposure all influence FTIR spectra and must be controlled or standardized to avoid misinterpretation. At the same time, the observed variability underscores the ecological and chemical richness of nectar, where both intrinsic plant metabolism and extrinsic environmental factors contribute to the spectral fingerprint.

## 3. Materials and Methods

### 3.1. Samples—Preparation and Collection

#### 3.1.1. Nectar and Plant Tissue Collection

##### *Echium vulgare* 

Flowers were selected in seven locations in Warsaw. At anthesis, before anther dehiscence, the entire nectar available in each flower was collected with microcapillary pipettes directly from the nectaries. Nectar from all flowers within one location was pooled and treated as a single sample. Whole flowering plants of *E. vulgare* were collected in one of the study populations and used for analysis without further modifications.

##### *Hedera helix* 

Flowers in six locations in Warsaw were selected. At anthesis, before anther dehiscence, the total nectar available in an inflorescence was sampled. Because in *H. helix* nectar accumulates on the floral nectary disc (sometimes referred to as a stylopodium), each disc within an inflorescence was rinsed with distilled water or 70% ethanol, and all liquid was collected using microcapillary pipettes. Nectar from all inflorescences within one location was pooled and treated as a single sample. While this step ensured sufficient collection volume, it may have introduced additional variance due to solvent–matrix interactions. This methodological limitation is discussed in Section 2.

#### 3.1.2. Artificial Samples

Artificial nectar samples were prepared by dissolving sucrose to obtain a 40% sugar solution in deionized water. The solutions were placed in plastic jars with wide necks and perforated lids. The holes allowed for the entry of airborne pollutants while preventing contamination by larger particles such as leaves or insects. The jars were positioned in two distinct locations in Warsaw, Poland: near a heavily trafficked and polluted bus stop, and in the garden, serving as a relatively pollution-free reference site. Samples were collected after 1, 2, 3, 4, and 5 months of exposure to allow sufficient time for the absorption or deposition of airborne pollutants. This experimental setup was designed to mimic potential environmental effects on real nectar and to facilitate the development of a classification model for environmental exposure.

### 3.2. IR Measurements

Spectra were acquired using a Nicolet 8700 FTIR spectrometer equipped with thermoelectrically cooled DLaTGS detector and Thermo Scientific Smart iTR accessory, provided by Thermo Fisher Scientific, Waltham, Massachusetts, U.S. In each ATR experiment a droplet of nectar/nectar-mimicking sample (previously dried at 50 °C for a week) was deposited on the surface of a germanium crystal and measured at room temperature. 64 scans at 4 cm^−1^ resolution were co-added to obtain the spectrum; each sample was measured 5 times.

### 3.3. Spectral Data Pre-Processing

A total of eight preprocessing variants were systematically evaluated to improve spectral quality and subsequent classification performance. The tested approaches included Savitzky–Golay smoothing and derivatives (window size = 11, polynomial order = 2), asymmetric least squares (ALS) baseline correction, normalization techniques such as Standard Normal Variate (SNV), MinMax scaling, and Z-score standardization, as well as Multiplicative Scatter Correction (MSC), which combines baseline and normalization functions. All preprocessing steps were implemented in a unified pipeline consisting of the following stages: (i) optional Savitzky–Golay smoothing or derivative transformation, (ii) selection of the fingerprint region (800–1500 cm^−1^) and the C–H stretching region (2750–3000 cm^−1^), (iii) baseline correction, and (iv) normalization. The performance of each variant was assessed using silhouette scores and classification accuracy metrics. All preprocessing and analyses were performed in the Google Colab environment.

### 3.4. Chemometric Analysis

The best-performing preprocessing variants were further examined using Principal Component Analysis (PCA). Two- and three-dimensional score plots were generated to provide a more detailed exploration of sample clustering. PCA loading plots were analyzed to identify the most discriminative spectral regions contributing to class separation. Clustering quality was quantified using silhouette scores to compare the effectiveness of each method in distinguishing between the nectar samples from *E. vulgare* and *H. helix* collected in different parts of the city of Warsaw, Poland as well as artificial nectar-mimicking samples. All chemometric analyses were performed in the Google Colab environment.

### 3.5. Use of GenAI

Generative artificial intelligence (GenAI) was used in this article to generate part of the code in Google Colab and to generate part of the introduction and abstract.

## 4. Conclusions

This study demonstrates the potential of FTIR spectroscopy combined with chemometric analysis as a powerful approach for nectar discrimination. Using *E. vulgare* and *H. helix* as model species, we obtained the following key results:

- Diagnostic spectral regions: the carbohydrate fingerprint (1200–950 cm^−1^) and the C–H stretching zone (2935–2885 cm^−1^) consistently provided the most discriminative information, confirming previous findings that carbohydrate-related bands dominate nectar and honey spectra.
- Preprocessing strategies: among eight tested variants, simple approaches such as Savitzky–Golay smoothing or even raw spectra achieved the highest clustering quality. More complex preprocessing, especially high-order derivatives, did not improve discrimination and often reduced performance.
- Multivariate analysis: PCA revealed that the majority of variance is explained by carbohydrate-associated bands. While PC1 and PC2 captured strong contributions from ~1050–1000, 980, and 930 cm^−1^, PC3 highlighted subtler variance in CH-stretching bands, refining separation between closely related sites.
- Collection strategy: the comparison of nectar collected in different solvents (water vs. ethanol) and matrices (nectar vs. floral tissue) demonstrated that methodological choices critically affect reproducibility and interpretation. Rinsing nectary discs in *H. helix* ensured sufficient volume but introduced additional variance, whereas nectar samples of E. vulgare provided cleaner carbohydrate-dominated fingerprints. Artificial models confirmed that environmental exposure also leaves a detectable chemical imprint on nectar-like matrices.
- Ecological and applied relevance: the approach captures location-dependent variability even within a single city, underscoring its utility for ecological studies of plant–pollinator interactions. At the same time, the method is directly applicable to food authentication, honey quality assessment, and the detection of adulteration.

Overall, FTIR spectroscopy offers a rapid, non-destructive, and reproducible tool for analyzing nectar composition. When combined with straightforward preprocessing and multivariate analysis, it provides a robust framework for characterizing both intrinsic plant chemistry and extrinsic environmental influences.

## Figures and Tables

**Table 1:** Main FTIR bands observed in nectar and honey samples, their assignments, functional group vibrations, and notes on typical sugar-specific markers.

Wavenumber (cm^−1^)	Assignment	Functional Group/Vibration	Notes/Typical Sugar Marker
~3700–3000	ν(O–H) broad	Hydroxyl groups, water, carbohydrates	Often max near 3300 cm^−1^, overlaps with H-bonded acids
~3050–2970	ν(C–H) asym	CH groups	Aliphatic C–H stretching, less intense in sugars
2970–2930	ν_as(CH_2_/CH_3_)	CH_2_, CH_3_	Characteristic in saccharide backbones
2935–2885	ν_s(CH_2_/CH_3_)	CH_2_, CH_3_	Major carbohydrate CH bands, diagnostic intensity
~1730–1600	ν(C=O), δ(O–H), δ(N–H)	Carbonyls, water, proteins	C=O stretch of fructose (ketone) and glucose (aldehyde), Amide I
~1640	δ(H–O–H)	Water	Overlaps with organics
1470–1360	δ(CH), δ(O–H)	CH/OH deformation	Sugars, organic acids
1366–1339	δ(O–H), δ(C–H)	Carbohydrates	Peak ~1347 cm^−1^ assigned to C–OH bending
1245–1150	ν(C–H), ν(C–O)	Sugars	Contributes to fingerprint differences
1175–1149	ν(C–O)	Sucrose marker	Often diagnostic for disaccharides
1095	ν(C–C), ν(C–O), δ(C–H)	Fructose	Typical strong absorption
1053	ν(C–O), δ(C–O–H)	Fructose	Prominent band
1032	ν(C–C), δ(C–H)	Glucose	Glucose-specific absorption
1027–958	ν(C–O), ν(C–C)	General carbohydrates	C–OH or C–C stretching
982	ν(C–H), ν(C–C)	Sucrose marker	Sometimes overlaps with glucose
983, 965	ν(C–O)	Fructose	Characteristic pair
950–750	Anomeric region	C–O–C linkages, δ(C–H)	Sensitive to glycosidic configuration
892	δ(C–H) anomeric	Carbohydrates	Reported as marker band in nectar honeys
776	δ(C–H), ring modes	Sugars	C–O–H and C–C–H bending

## Data Availability

Data are available on request from the corresponding author.

## Supplementary Materials

The following supporting information can be downloaded at: https://www.mdpi.com/article/10.3390/molecules30204083/s1. Table S1. Silhouette scores obtained for different preprocessing strategies applied to *E. vulgare* and *H. helix* nectar spectra. Higher values indicate better clustering quality; Table S2. Summary of preprocessing strategies and parameters used for ATR-FTIR spectra of nectar samples; Figure S1. Map with the locations of nectar samples of *E. vulgare* (navy blue) and *H. helix* (brown); Figure S2. ATR-FTIR spectra of fructose, glucose and sucrose recorded to facilitate spectral analysis of nectars; Figure S3. PCA loadings of *E. vulgare* nectar spectra after Savitzky–Golay smoothing, restricted to the 800–1500 and 2750–3000 cm^−1^ regions. The first three components are shown: (a) PC1 explaining 59.4% of the total variance, (b) PC2 explaining 38.6%, and (c) PC3 explaining 1.3%.; Figure S4. PCA loadings of *H. helix* nectar spectra after baseline correction and MinMax normalization, restricted to the 800–1500 and 2750–3000 cm^−1^ regions. The first three components are shown: (a) PC1 explaining 62.1% of the total variance, (b) PC2 explaining 23.9%, and (c) PC3 explaining 6.8%.; Figure S5. Average raw FT-IR spectra (±SD) of nectar samples collected from clean and dirty lo-cations, compared with control samples. Spectra are shown across the full 4000–500 cm^−1^ range.

## Abbreviations

The following abbreviations are used in this manuscript:

ATR	Attenuated Total Reflectance
ALS	Asymmetric Least Squares (baseline correction)
CCA	Common Component Analysis
DLLME	Dispersive Liquid–Liquid Microextraction
DHA	Dihydroxyacetone
FAAS/FAES	Flame Atomic Absorption/Emission Spectrometry
FTIR	Fourier Transform Infrared Spectroscopy
FT-Raman	Fourier Transform Raman Spectroscopy
GC-MS	Gas Chromatography–Mass Spectrometry
GenAI	Generative Artificial Intelligence
HCA	Hierarchical Cluster Analysis
HMF	Hydroxymethylfurfural
HPLC	High-Performance Liquid Chromatography
ICP-MS	Inductively Coupled Plasma Mass Spectrometry
ICP-OES	Inductively Coupled Plasma Optical Emission Spectrometry
IR	Infrared
LC-MS/LC-MS/MS	Liquid Chromatography–Mass Spectrometry/tandem LC-MS
MIR	Mid-Infrared Spectroscopy
MSC	Multiplicative Scatter Correction
MSPE	Magnetic Solid-Phase Extraction
NIR	Near-Infrared Spectroscopy
PCA	Principal Component Analysis
PLS/PLSR/PLS-DA	Partial Least Squares/Partial Least Squares Regression/Partial Least Squares Discriminant Analysis
QuEChERS	Quick, Easy, Cheap, Effective, Rugged, Safe (extraction method)
SERS	Surface-Enhanced Raman Spectroscopy
SD	Standard deviation
SG	Savitzky–Golay (smoothing/derivatives)
SNV	Standard Normal Variate
SPE	Solid-Phase Extraction
SVM	Support Vector Machine
TLC	Thin Layer Chromatography

## References

[1] Pohl P., Stecka H., Sergiel I., Jamroz P. (2012). Different Aspects of the Elemental Analysis of Honey by Flame Atomic Absorption and Emission Spectrometry: A Review. Food Anal. Methods.

[2] Jovetić M.S., Redžepović A.S., Nedić N.M., Vojt D., Đurđić S.Z., Brčeski I.D., Milojković-Opsenica D.M. (2018). Urban Honey—The Aspects of Its Safety. Arch. Ind. Hyg. Toxicol..

[3] Nicolson S.W. (2022). Sweet Solutions: Nectar Chemistry and Quality. Philos. Trans. R. Soc. B.

[4] Flores Ortiz C.M., Castro I.P., Portilla L.B.H., Aranda P.D.D., Arizmendi M.D.C. (2003). Carbohydrate Analysis of Floral Nectar Using Medium Infrared. Phytochem. Anal..

[5] Nickless E.M., Holroyd S.E., Hamilton G., Gordon K.C., Wargent J.J. (2016). Analytical Method Development Using FTIR-ATR and FT-Raman Spectroscopy to Assay Fructose, Sucrose, Glucose and Dihydroxyacetone, in *Leptospermum scoparium* Nectar. Vib. Spectrosc..

[6] Bicudo De Almeida-Muradian L., Monika Barth O., Dietemann V., Eyer M., Freitas A.D.S.D., Martel A.-C., Marcazzan G.L., Marchese C.M., Mucignat-Caretta C., Pascual-Maté A. (2020). Standard Methods for Apis Mellifera Honey Research. J. Apic. Res..

[7] Lo Turco V., Di Bella G., Potortì A.G., Tropea A., Casale E.K., Fede M.R., Dugo G. (2016). Determination of Plasticisers and BPA in Sicilian and Calabrian Nectar Honeys by Selected Ion Monitoring GC/MS. Food Addit. Contam. Part A.

[8] Farajzadeh M.A., Safi R., Yadeghari A. (2019). Combination of QuEChERS Extraction with Magnetic Solid Phase Extraction Followed by Dispersive Liquid–Liquid Microextraction as an Efficient Procedure for the Extraction of Pesticides from Vegetable, Fruit, and Nectar Samples Having High Content of Solids. Microchem. J..

[9] Orso D., Martins M.L., Donato F.F., Rizzetti T.M., Kemmerich M., Adaime M.B., Zanella R. (2014). Multiresidue Determination of Pesticide Residues in Honey by Modified QuEChERS Method and Gas Chromatography with Electron Capture Detection. J. Braz. Chem. Soc..

[10] Tu X., Chen W. (2021). Overview of Analytical Methods for the Determination of Neonicotinoid Pesticides in Honeybee Products and Honeybee. Crit. Rev. Anal. Chem..

[11] Stecka H., Jedryczko D., Welna M., Pohl P. (2014). Determination of Traces of Copper and Zinc in Honeys by the Solid Phase Extraction Pre-Concentration Followed by the Flame Atomic Absorption Spectrometry Detection. Environ. Monit. Assess..

[12] Tibebe D., Hussen M., Mulugeta M., Yenealem D., Moges Z., Gedefaw M., Kassa Y. (2022). Assessment of Selected Heavy Metals in Honey Samples Using Flame Atomic Absorption Spectroscopy (FAAS), Ethiopia. BMC Chem..

[13] Miaw C.S.W., Assis C., Silva A.R.C.S., Cunha M.L., Sena M.M., De Souza S.V.C. (2018). Determination of Main Fruits in Adulterated Nectars by ATR-FTIR Spectroscopy Combined with Multivariate Calibration and Variable Selection Methods. Food Chem..

[14] Caramês E.T.S., Alamar P.D., Poppi R.J., Pallone J.A.L. (2017). Quality Control of Cashew Apple and Guava Nectar by near Infrared Spectroscopy. J. Food Compos. Anal..

[15] Folli G.S., Santos L.P., Santos F.D., Cunha P.H.P., Schaffel I.F., Borghi F.T., Barros I.H.A.S., Pires A.A., Ribeiro A.V.F.N., Romão W. (2022). Food Analysis by Portable NIR Spectrometer. Food Chem. Adv..

[16] Gok S., Severcan M., Goormaghtigh E., Kandemir I., Severcan F. (2015). Differentiation of Anatolian Honey Samples from Different Botanical Origins by ATR-FTIR Spectroscopy Using Multivariate Analysis. Food Chem..

[17] Prata J.C., da Costa P.M. (2024). Fourier Transform Infrared Spectroscopy Use in Honey Characterization and Authentication: A Systematic Review. ACS Food Sci. Technol..

[18] Etzold E., Lichtenberg-Kraag B. (2008). Determination of the Botanical Origin of Honey by Fourier-Transformed Infrared Spectroscopy: An Approach for Routine Analysis. Eur. Food Res. Technol..

[19] Liberda D., Pięta E., Pogoda K., Piergies N., Roman M., Koziol P., Wrobel T.P., Paluszkiewicz C., Kwiatek W.M. (2021). The Impact of Preprocessing Methods for a Successful Prostate Cell Lines Discrimination Using Partial Least Squares Regression and Discriminant Analysis Based on Fourier Transform Infrared Imaging. Cells.

[20] Lichtenberg-Kraag B., Hedtke C., Bienefeld K. (2002). Infrared Spectroscopy in Routine Quality Analysis of Honey. Apidologie.

[21] Kędzierska-Matysek M., Matwijczuk A., Florek M., Barłowska J., Wolanciuk A., Matwijczuk A., Chruściel E., Walkowiak R., Karcz D., Gładyszewska B. (2018). Application of FTIR Spectroscopy for Analysis of the Quality of Honey. BIO Web Conf..

[22] Matwijczuk A., Budziak-Wieczorek I., Czernel G., Karcz D., Barańska A., Jedlińska A., Samborska K. (2022). Classification of Honey Powder Composition by FTIR Spectroscopy Coupled with Chemometric Analysis. Molecules.

[23] Bunaciu A.A., Aboul-Enein H.Y. (2022). Honey Discrimination Using Fourier Transform-Infrared Spectroscopy. Chemistry.

[24] Shiddiq M., Zulkarnain, Asyana V., Aliyah H. (2019). Identification of Pure and Adulterated Honey Using Two Spectroscopic Methods. J. Phys. Conf. Ser..

[25] Damto T., Zewdu A., Birhanu T. (2023). Application of Fourier Transform Infrared (FT-IR) Spectroscopy and Multivariate Analysis for Detection of Adulteration in Honey Markets in Ethiopia. Curr. Res. Food Sci..

[26] David M., Berghian-Grosan C., Magdas D.A. (2025). Honey Differentiation Using Infrared and Raman Spectroscopy Analysis and the Employment of Machine-Learning-Based Authentication Models. Foods.

[27] Cozzolino D., Corbella E., Smyth H.E. (2011). Quality Control of Honey Using Infrared Spectroscopy: A Review. Appl. Spectrosc. Rev..

[28] Farber C., Li J., Hager E., Chemelewski R., Mullet J., Rogachev A.Y., Kurouski D. (2019). Complementarity of Raman and Infrared Spectroscopy for Structural Characterization of Plant Epicuticular Waxes. ACS Omega.

[29] González Fá A., Pignanelli F., López-Corral I., Faccio R., Juan A., Di Nezio M.S. (2019). Detection of Oxytetracycline in Honey Using SERS on Silver Nanoparticles. TrAC Trends Anal. Chem..

[30] Fodor M., Matkovits A., Benes E.L., Jókai Z. (2024). The Role of Near-Infrared Spectroscopy in Food Quality Assurance: A Review of the Past Two Decades. Foods.

[31] Wiercigroch E., Szafraniec E., Czamara K., Pacia M.Z., Majzner K., Kochan K., Kaczor A., Baranska M., Malek K. (2017). Raman and Infrared Spectroscopy of Carbohydrates: A Review. Spectrochim. Acta Part A Mol. Biomol. Spectrosc..

[32] Grzybek M., Kukula-Koch W., Strachecka A., Jaworska A., Phiri A.M., Paleolog J., Tomczuk K. (2016). Evaluation of Anthelmintic Activity and Composition of Pumpkin (*Cucurbita pepo* L.) Seed Extracts—In Vitro and in Vivo Studies. Int. J. Mol. Sci..

