# Supplementary material for: Data-Driven FTIR Spectroscopy for the Discrimination of Nectars
Source: Molecules. 2025 Oct 14;30(20):4083. doi: 10.3390/molecules30204083 (PMC12565982; doi:10.3390/molecules30204083)
Supplement: Supplementary file 1 [file molecules-30-04083-s001.zip › molecules-3898819-supplementary.pdf]

Table S1. Silhouette scores obtained for different preprocessing strategies applied to Echium vulgare and Hedera helix nectar spectra. Higher values indicate better clustering quality.

**Preprocessing**	**Silhouette score (*E. vulgare*)**	**Silhouette score (*H. helix*)**
SG smooth	0.836298	0.141356
Raw	0.835962	0.141323
SG + Baseline + SNV	0.828089	0.386285
Baseline	0.826326	0.270291
Baseline + MSC	0.823453	0.373550
Baseline + SNV	0.823357	0.385520
Baseline + Zscore	0.823357	0.385520
Baseline + MinMax	0.822137	0.394844
SG 1st	0.782165	0.257049
SG 2nd	0.467806	0.223667

Table S2. Summary of preprocessing strategies and parameters used for ATR-FTIR spectra of nectar samples.

**Preprocessing variant**	**Baseline correction**	**Normalization**	**Savitzky–Golay (SG) parameters**	**Notes**
Raw spectra	–	–	–	No preprocessing; restricted to 800–1500 and 2750–3000 cm^{-1}
SG smooth	–	–	Window = 11, Polynomial = 2	Applied to full spectrum before range selection
SG + Baseline + SNV	ALS baseline	SNV	Window = 11, Polynomial = 2	Best clustering for *H. helix*
Baseline only	ALS baseline	–	–	Corrects scattering/background
Baseline + MSC	ALS baseline	MSC	–	MSC applied after baseline
Baseline + SNV	ALS baseline	SNV	–	–
Baseline + Z-score	ALS baseline	Z-score	–	–
Baseline + Min–Max	ALS baseline	Min–Max	–	–
SG 1st derivative	–	–	Window = 11, Polynomial = 2, Derivative = 1	Highlights peak shifts
SG 2nd derivative	–	–	Window = 11, Polynomial = 2, Derivative = 2	Amplifies noise, lowest silhouette

ALS – Asymmetric Least Squares baseline correction; MSC – Multiplicative Scatter Correction; SNV – Standard Normal Variate.

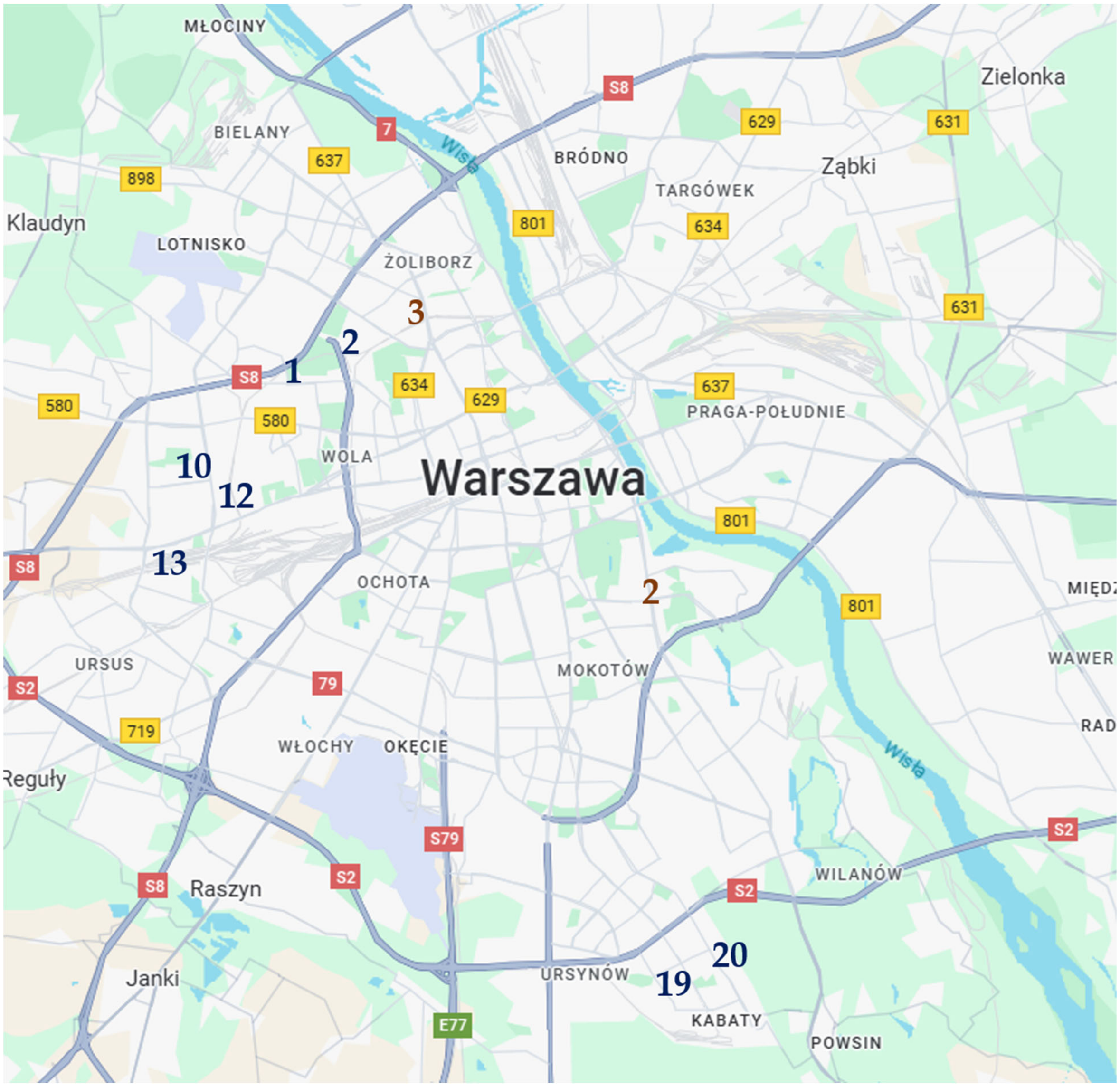

Figure S1. Map with the locations of nectar samples of *E. vulgare* (navy blue) and *H. helix* (brown).

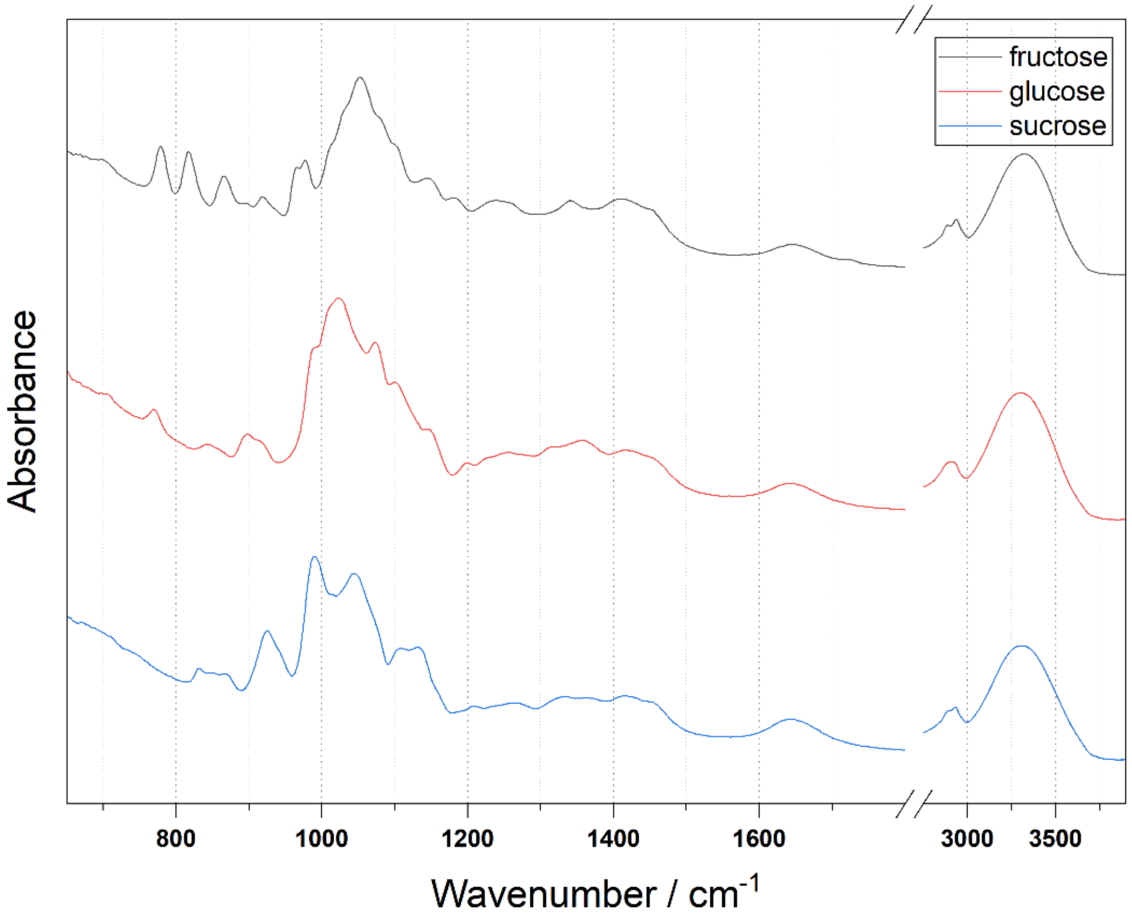

Figure S2. ATR-FTIR spectra of fructose, glucose and sucrose recorded to facilitate spectral analysis of nectars.

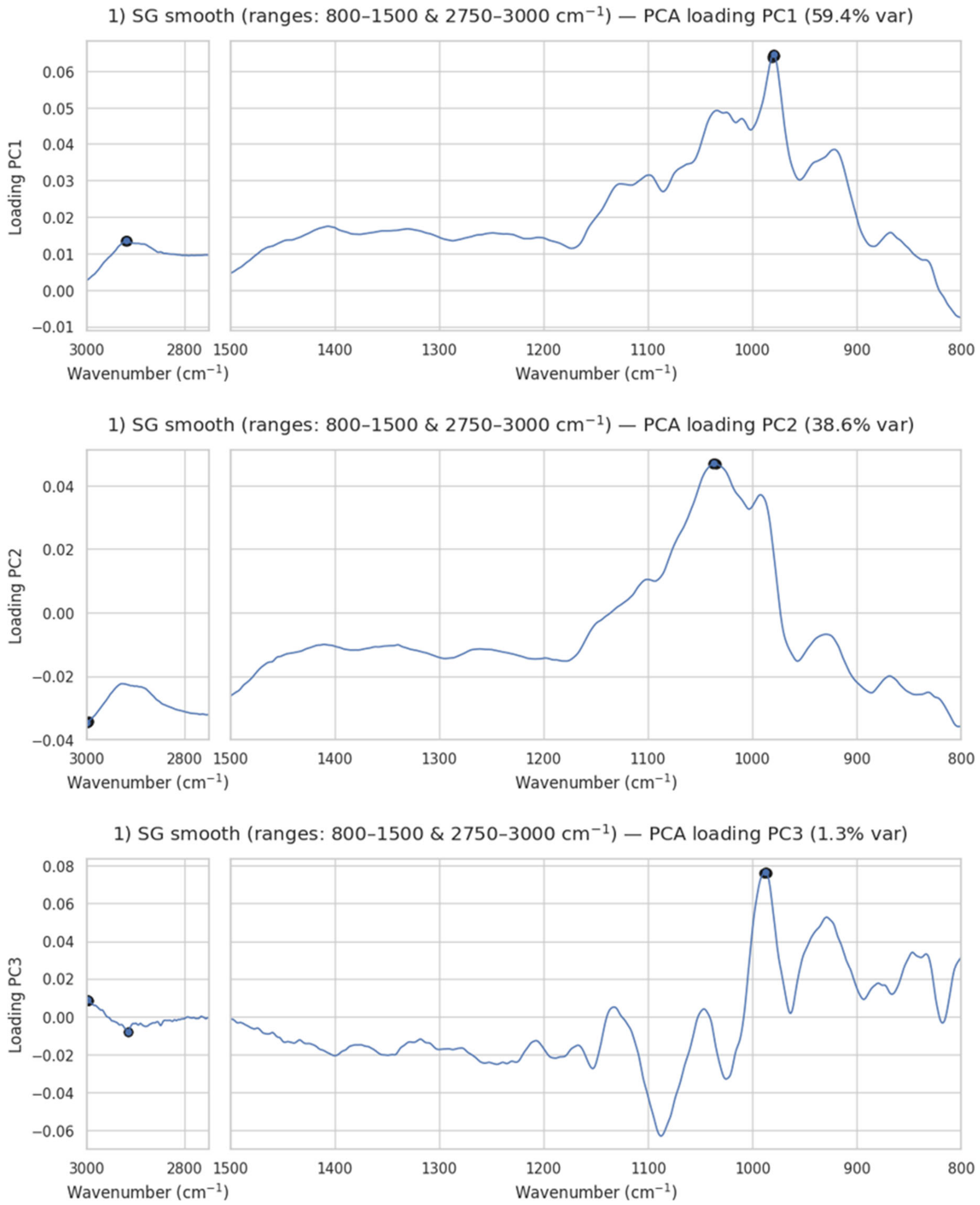

Figure S3. PCA loadings of *Echium vulgare* nectar spectra after Savitzky–Golay smoothing, restricted to the 800–1500 and 2750–3000 cm^{-1} regions. The first three components are shown: (a) PC1 explaining 59.4% of the total variance, (b) PC2 explaining 38.6%, and (c) PC3 explaining 1.3%.

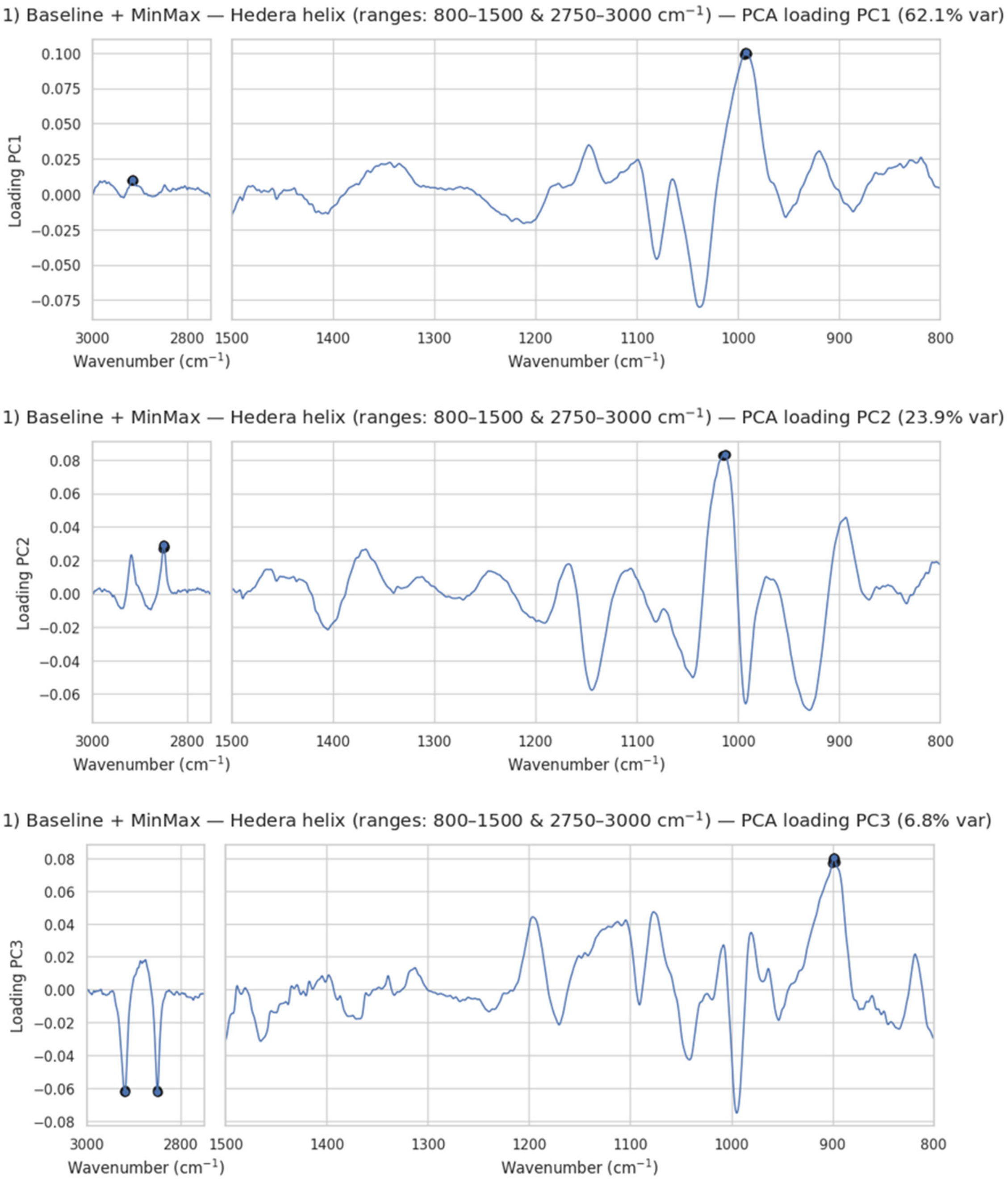

Figure S4. PCA loadings of Hedera helix nectar spectra after baseline correction and MinMax normalization, restricted to the 800–1500 and 2750–3000 cm^{-1} regions. The first three components are shown: (a) PC1 explaining 62.1% of the total variance, (b) PC2 explaining 23.9%, and (c) PC3 explaining 6.8%.

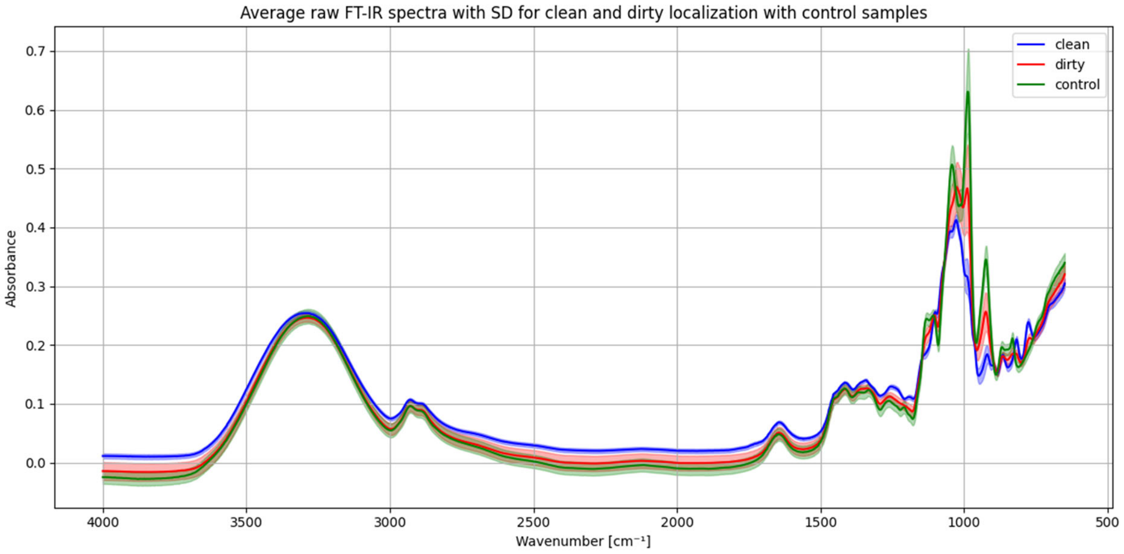

Figure S5. Average raw FT-IR spectra (±SD) of nectar samples collected from clean and dirty locations, compared with control samples. Spectra are shown across the full 4000–500 cm^{-1} range.
